# A systematic review of metacognitions in Internet Gaming Disorder and problematic Internet, smartphone and social networking sites use

**DOI:** 10.1002/cpp.2588

**Published:** 2021-05-04

**Authors:** Silvia Casale, Alessia Musicò, Marcantonio M. Spada

**Affiliations:** ^1^ Department of Health Sciences, Psychology Unit University of Florence Florence Italy; ^2^ Department of Experimental and Clinical Medicine University of Florence Florence Italy; ^3^ Division of Psychology, School of Applied Sciences London South Bank University London UK

**Keywords:** Internet Gaming Disorder, metacognitions, problematic Internet use, problematic smartphone use, problematic social networking sites use, systematic review

## Abstract

**Background:**

The use of new technologies is growing, and some authors have suggested that frequent use might hide a non‐chemical addiction (i.e., technological addiction). Over the last 5 years, several studies investigating the role of metacognitions in technological addictions have been published. We aim to provide the first systematic review focused on this topic, by updating the initial evidence highlighted by a previous systematic review on metacognitions across addictive behaviours (Hamonniere & Varescon, 2018).

**Methods:**

Electronic literature databases (Pubmed, PsychINFO, SCOPUS and Web of Science) were searched to identify studies that examined the relationship between metacognitions and four different technological addictions (Internet Gaming Disorder, IGD; problematic Internet use, PIU; problematic smartphone use, PSU; and problematic social networking sites use, PSNSU).

**Results:**

We found 13 empirical studies published between 2018 and 2021. Positive low to moderate cross‐sectional associations between the four technological addictions and both generic and specific metacognitions were found, in accordance with the metacognitive model of addictive behaviours. Positive beliefs about worry, negative beliefs about thoughts concerning uncontrollability and danger, beliefs about the need to control thoughts and a lack of cognitive confidence were associated with IGD, PIU, PSU and PSNSU.

**Conclusions:**

The absence of longitudinal studies prevents us from providing definitive answers about the role of metacognitions in technological addictions. Despite this limitation, interventions that target metacognitions could be beneficial for people presenting with technological addictions.

**Key Practitioner Message**

- Results showed positive associations between metacognitions and technological addictions.
- Metacognitions should be considered during the clinical assessment process.
- Interventions that target metacognitions could be beneficial for individuals presenting with problematic technological use.

## 1 INTRODUCTION

Technology‐related impacts are an area of particular interest for the speed with which new technology is implemented. The use of new technologies is growing, especially, but not only, among young people. Fifty percent of the world population use the Internet, and more than 3.8 billion people (i.e., almost 49% of the world's population) own a smartphone (Statista, 2021). The daily time mobile phone users spent using their devices rose from 152 min in 2014 to 215 min in 2018 and is expected to grow to 234 min by 2021. The number of mobile devices operating worldwide was 14.02 billion in 2020, and it is expected to reach 17.72 billion by 2024, an increase of 3.7 billion devices compared to 2020 levels (Statista, 2021). In 2020, over 3.6 billion people were using social media worldwide, a number projected to increase to almost 4.41 billion in 2025.

This rapid growth in popularity of new technologies has led to various theoretical discussions and empirical investigations on the potential benefits of their use. Online social media and gaming, for instance, represent an important developmental context for the handling of certain issues, which are characteristic of adolescence, such as gender and identity exploration, self‐expression and the need for peer acceptance (Gerwin et al., 2018). Online games seem to have great positive therapeutic potential in addition to their entertainment value (Griffiths et al., 2017), whilst smartphone apps provide a non‐invasive, inexpensive and easy to use solution for clinicians. Despite various advantages, the lack of self‐regulation in the use of the various new technologies has been well documented, and overuse of digital technologies has been recognized as a public health concern (World Health Organization, 2015). Some authors (e.g., Griffiths, 1995) have raised the possibility that frequent use might hide a non‐chemical addiction, which involves human–machine interaction (i.e., technological addiction). From this viewpoint, technological addictions could be considered a subset of behavioural addictions that are characterized by the six core dimensions of the components model of addiction (Griffiths, 2005): salience, tolerance, conflict, mood modification, withdrawal and relapse. Given the current debate on the topic (see, for a discussion, Montag et al., 2021; Starcevic et al., 2020), a further description of what we meant by technological addiction is needed. We argue that the concept of technological addiction makes sense only if the use of technology is essential for the addiction development. In other words, to define a behavioural addiction as a technological addiction, technology should not be a mere vehicle or a means to access the object of the addiction. For instance, the use of technology is not an essential feature of gambling—similarly, it is not an essential feature for shopping addiction or pornography addiction, because it is plausible to suppose that these dependencies would exist in the absence of technology and/or the Internet (Caplan, 2002; Casale et al., 2014; Davis, 2001). Conversely, in Internet Gaming Disorder (IGD), problematic smartphone use (PSU) and problematic social networking sites use (PSNSU), the utilization of technology is a necessary component of the addiction itself.

That said, some authors have suggested that excessive technology use might reflect a temporary compensatory strategy to cope with transient negative states rather than a pervasive stable pattern of behaviour (see Billieux et al., 2015; Carbonell & Panova, 2017; Kardefelt‐Winther, 2014). Despite these conflicting positions, the empirical literature has shown that IGD, PSU and PSNSU share some core features with established addictions, suggesting that excessive technology use deserves scientific attention. Existing empirical evidence has thus far highlighted the commonality between the neural mechanisms underlying substance use disorder and IGD (e.g., Fauth‐Bühler & Mann, 2017), PSU (e.g., Horvath et al., 2020) and PSNSU (e.g., Aydın, Obuća, et al., 2020; Lee et al., 2021). In keeping with these results, craving symptoms have been reported among IGD subjects (e.g., King et al., 2016), social media users (e.g., Stieger & Lewetz, 2018) and smartphone users (e.g., Wilcockson et al., 2019) under abstinence conditions. Withdrawal effects across technological addictions have also been highlighted through various experimental studies (see, for a review, Fernandez et al., 2020). Similar results have been observed by those empirical studies that have used the umbrella category of problematic Internet use (PIU; Niu et al., 2016; Wang et al., 2017).

Over the past two decades, the concept of metacognition and its link with psychological problems has received increasing attention in clinical psychology. Flavell (1978, 1979) introduced the term ‘metacognitive knowledge’ defining it as knowledge about one's own (or someone else's) cognitions, motivations and emotions. Fifteen years later, Wells and Matthews (1994) introduced the Self‐Regulatory Executive Function (S‐REF) model of psychopathology, the first metacognitive model of psychopathology, which assigned a central role to metacognitions in the genesis of psychological dysfunction. They proposed that beliefs about cognitive‐affective experiences and ways of controlling these cognitive‐affective experiences (termed ‘metacognitions’ and also referred to as ‘metacognitive beliefs’) are involved in the activation and maintenance of maladaptive coping styles (rumination, worry and threat monitoring) that maintain psychological dysfunction. Five dimensions of generic metacognitions have been found to be involved in the preservation of maladaptive coping (Cartwright‐Hatton & Wells, 1997; Wells & Cartwright‐Hatton, 2004): (i) the belief that worrying helps to solve problems (i.e., positive beliefs about worry); (ii) the belief that thoughts may be uncontrollable and dangerous but need to be controlled in order to allow functioning (i.e., negative beliefs about thoughts concerning uncontrollability and danger); (iii) the belief that one's own cognitive skills—in particular, memory and attentional functioning—are ineffective (i.e., cognitive confidence); and (iv) the belief about the need to control thoughts; and (v) the degree to which an individual focuses on their own thinking processes (i.e., cognitive self‐consciousness).

Findings from empirical research have confirmed the transdiagnostic feature of generic metacognitions but also been highlighted that metacognitions might vary across disorders. For instance, positive beliefs about worry are common to depression and all anxiety disorders, whilst beliefs about the need to control thoughts and beliefs about thoughts influencing harm outcomes are prevalent in obsessive–compulsive disorder (OCD; Sun et al., 2017; Wells & Matthews, 1996).

In the field of addictive behaviours, metacognitions are conceptualized across three temporal phases: pre‐engagement, engagement and post‐engagement (Spada et al., 2013; Spada & Wells, 2009). Depending on their content, they can be separated into two factors: positive and negative metacognitions. Positive metacognitions refer to the benefits of engaging in a specific behaviour as a cognitive and affective self‐regulation strategy (e.g., ‘using my Smartphone will help me relax’). These metacognitions have been found to play a central role in the pre‐engagement phase because they motivate individuals to engage in addictive behaviour. Negative metacognitions concern the uncontrollability and dangers of thoughts and outcomes relating to the addictive behaviour employed (e.g., ‘thoughts about using my Smartphone interfere with my functioning’) and are activated in the engagement and post‐engagement phases. Because they trigger negative emotional states (Caselli et al., 2018; Spada, Caselli, et al., 2015), these metacognitions are thought to be involved in the perpetuation of addictive behaviours as a means of regulating these internal states. Empirical evidence supports the role of generic metacognitions and positive and negative metacognitions specific to addictive behaviours, in substance‐based addictive behaviours (Spada, Nikčević, et al., 2007; Spada & Wells, 2005; Spada, Zandvoort, & Wells, 2007) and recognized behavioural addictions, that is, gambling (e.g., Caselli et al., 2018; Jauregui et al., 2016; Spada, Giustina, et al., 2015). Positive and negative metacognitions have also been found to be a stronger predictor than outcome expectancies (i.e., the anticipated reinforcing and punishing consequences related to engaging in a specific behaviour) in cigarette use and nicotine dependence (Nikčević et al., 2017) and drinking behaviour (Spada, Moneta, & Wells, 2007).

A relatively recent systematic review of the empirical literature on metacognitions in addictive behaviours (Hamonniere & Varescon, 2018) concluded that among the five dimensions of generic metacognitions, beliefs about the need to control thoughts are those most closely associated with addictive behaviours. Negative beliefs about thoughts concerning uncontrollability and danger, and a lack of cognitive confidence, also appear to be fairly common across addictive behaviours. Such a systematic review also included the few studies published on technological addictions at the date of the literature search (20 February 2018). Seven studies were identified: five were focused on PIU and one each on problematic Facebook use (PFU) and IGD. No studies had yet been published on the role of metacognitions in PSU and PSNSU, and evidence regarding PFU and problematic online gaming was only based on one study each. That said, the above‐mentioned systematic review showed that (i) generic metacognitions (e.g., positive beliefs about worry and negative beliefs about thoughts concerning uncontrollability and danger) are associated with PIU and problematic online gaming and (ii) metacognitions specific to addictive behaviours deserve additional scientific attention (Hamonniere & Varescon, 2018).

### 1.1 The present review

Identifying metacognitions associated with problematic technology use may have important clinical implications for the treatment of maladaptive behaviour patterns involving human–machine interaction. Consequently, the present review aims to provide a systematic overview of the existing evidence on generic and specific metacognitions in technological addictions by considering the studies published after Hamonniere and Varescon's (2018) review.

## 2 METHODS

**FIGURE 1:**
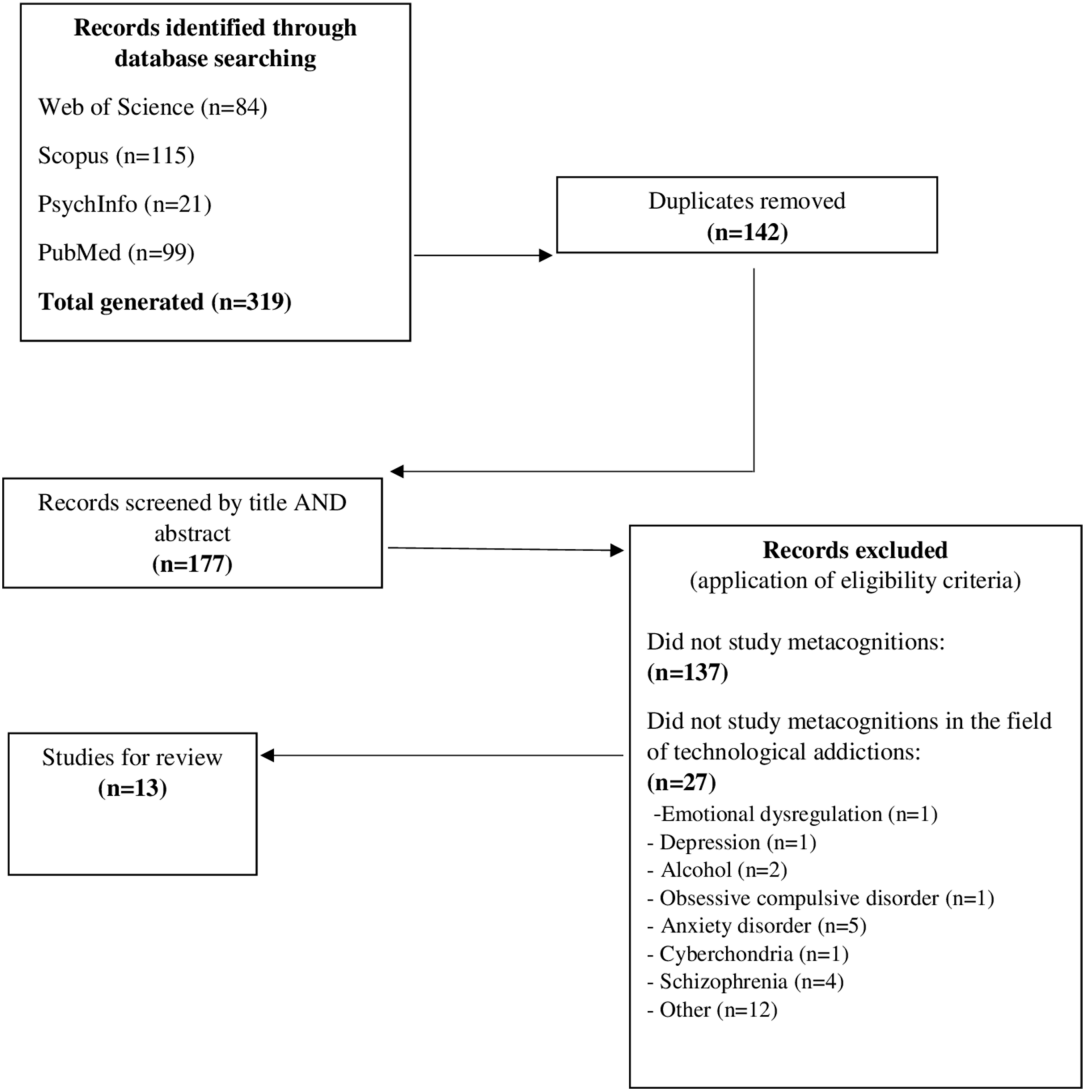
Search strategy

This study follows the Preferred Reporting Items for Systematic Reviews and Meta‐Analyses (PRISMA) guidelines (Moher et al., 2009). Studies were included if they (a) reported empirical findings on the relationship between metacognitions, as conceptualized in the S‐REF model and technological addictions (e.g., PIU or/and IGD or/and PSU or/and PSNSU), (b) were published in peer‐reviewed scholarly journals and were written in English and (c) were published between March 2018 and January 2021. The search strategy is detailed in Figure 1.

The following databases were searched in January 2021: PsycINFO, PubMed, Scopus and Web of Science. The latest search was run on 31 January 2021. A search string or subject term related to metacognitions (metacogniti*) was combined with a technological addiction‐related search string or subject term using Boolean operators. In detail, the following combinations were included: metacogniti* AND (addict* OR compuls* OR excess* OR problematic OR pathologic* OR disorder) AND (technolog* OR Internet OR computer OR smartphone OR mobile OR social media use OR social networking sites use OR Facebook OR game). The following procedure was used to assess the eligibility of the studies: title screening, abstract screening and full papers screening. The titles and abstracts of each article identified were screened by two researchers (S. C. and A. M.), and articles that according to both reviewers did not appear to meet the inclusion criteria were excluded. A total of 13 articles met these inclusion criteria. As all the 13 studies used a cross‐sectional design, we conducted the quality assessment of the 13 studies using the AXIS tool, a quality assessment tool for observational cross‐sectional studies (Downes et al., 2016). The tool comprises 21 items for which there are three response options (‘yes’, ‘no’ or ‘do not know’) to assess study quality and reporting transparency (with ‘*yes*’ scored as 1 and ‘*no*’ or ‘*do not know*’ scored as 0). As the interpretation of the scores is subjective, we used the following guidelines (Moor & Anderson, 2019): scores indicating *low quality* = 1–7; scores indicating *medium quality* = 8–14; and scores indicating *high quality* = 15–20). A quality score out of 20 is then generated. Table 1 shows the quality score for each study identified by this systematic review, and additional comments have been provided in Section 3.

**TABLE 1:** Overview of included studies

Authors	Country	Technology addiction	Metacognitions	Sample	Measures	Findings	Quality rating/20
Caselli et al. (2020)	Italy	PIU	Specific metacognitions to PIU	*n* = 326 online gamers (*M* = 93.3%) *M*_age_ = 27 (5.65)	*Metacognitions:* MOGS (Spada & Caselli, 2017) *Problematic Internet Use*: IAT (Young, 1998) *Negative affect:* HADS (Zigmond & Snaith, 1983)	‐Significant bivariate correlations between PIU and both positive metacognitions (*r* = 0.24**) and negative metacognitions (*r* = 0.36**) ‐Positive metacognitions about online gaming mediate the association between negative affect and weekly online gaming hours ‐Negative metacognitions mediate the association between weekly online gaming hours and PIU ‐Positive metacognitions predicted negative metacognitions through weekly online gaming hours	15
Hamidi and Ghasedi (2020)	Iran	PIU	Generic metacognitions	*n* = 60 Internet addicted; 60 drug addicted; 60 non‐addicted *M*_age_ = n.r. (age range: 18–30)	Metacognitions: MCQ‐30 (Wells & Cartwright‐Hatton, 2004) *Problematic Internet use:* IAT (Young, 1998)	Bivariate correlations: n.r. ‐Internet addicted obtained significant higher scores than controls in negative beliefs about thoughts concerning uncontrollability and danger, and cognitive confidence	5
Hashemi et al. (2020)	Iran	PIU	Generic metacognitions	*n* = 651 community participants (*F* = 62.5%) *M*_age_ = 33.53 (*SD* = 10.81; age range = 13–73 years)	*Metacognitions*:MCQ‐30 (Wells & Cartwright‐Hatton, 2004) *Problematic Internet use:* GPIUS (Caplan, 2010)	Significant bivariate correlations between PIU and positive beliefs about worry (*r* = 0.34***), negative beliefs about thoughts concerning uncontrollability and danger (*r* = 0.48**), beliefs about the need to control thoughts (*r* = 0.42**), cognitive confidence (*r* = 0.43**), and cognitive monitoring (*r* = 0.13**).	15
Marci et al. (2021)	Italy	PIU	Generic metacognitions	*n = 538 early adolescents* *(F = 51%)* *M*_*age*_ = 12.7 (*SD* = 0.87; age range = 10–14 years)	*Metacognitions:* MCQ‐C (Bacow et al., 2009) *Problematic Internet use:* SPIUT (Siciliano et al., 2015)	‐Significant bivariate correlation between negative meta‐worry and PIU (*r* = 0.20***). ‐Negative meta‐worry mediates the association between anxiety towards mother and avoidance towards father with PIU	15
Aydın, Güçlü, et al. (2020)	Turkey	IGD	Generic metacognitions	*n* = 477 eighth‐grade secondary school students (*F* = 47%) *M*_age_ = 13.21 (*SD* = 0.56)	*Metacognitions:* MCQ‐C (Bacow et al., 2009) *Internet Gaming Disorder:* IGD‐T (Pontes et al., 2014) *Emotion recognition:* RMET (Baron‐Cohen et al., 2001)	‐Significant bivariate correlations between IGD total score and positive meta‐worry (*r* = 0.22**), negative meta‐worry (*r* = 0.23**), beliefs about the need to control thoughts (*r* = 0.23**) and cognitive monitoring (*r* = 0.16**). After controlling for daily Internet use and negative emotion recognition: ‐Positive meta‐worry independently predict salience, tolerance, conflict, relapse and the IGD‐T total score ‐Negative meta‐worry independently predict withdrawal conflict and the IGD‐T total score ‐Cognitive monitoring predicted mood modification	16
Marino et al. (2020)	Italy	IGD	Specific metacognitions to IGD	*n* = 543 online gamers (*F* = 17.5%) *M*_age_ = 23.9 years (*SD* = 6.15)	*Metacognitions*: MOGS (Spada & Caselli, 2017) *Internet Gaming Disorder:* IGDS9‐SFT (Pontes & Griffiths, 2015) *Social anxiety:* I‐SPIN (Connor et al., 2000)	‐Significant bivariate correlations between IGD and both positive metacognitions (*r* = 0.26**) and negative metacognitions (*r* = 0.64**) Positive and negative metacognition mediate the association between social anxiety and IGD scores.	15
Zhang et al. (2020)	China	IGD	Generic metacognitions	*n* = 680 university students (*F* = 69.1%) *M*_age_ = 19.72 (*SD* = 1.38)	*Metacognitions*: MCQ‐30 (Wells & Cartwright‐Hatton, 2004) *IGD tendency:* DSM 5 diagnostic criteria *Depression and anxiety* DASS‐21‐Chinese version (Wang et al., 2016)	Positive associations between IGD scores and positive beliefs about worry (*r* = 0.30***), negative beliefs about thoughts concerning uncontrollability and danger (*r* = 0.24***), cognitive confidence (*r* = 0.28***), beliefs about the need to control thoughts (r = 0.33***) and cognitive monitoring (*r* = 0.16***). Cognitive confidence had significant indirect effect on IGD tendency via depression after controlling for anxiety and demographics. Positive beliefs about worry, cognitive confidence, and cognitive monitoring had significant indirect effect on IGD tendency via anxiety, after controlling for depression and demographics	17
Balıkçı et al. (2020)	Bosnia and Herzegovina	PSNSU	Generic metacognitions	*n* = 308 college students Non‐addicts = 119 (*F* = 52.1%) *M*_age_ = 21.71 (*SD* = 2.24) SNS addicts = 189 (*F* = 59.26%) *M*_age_ = 21.08 (*SD* = 1.92)	*Metacognitions*: MCQ‐30 (Wells & Cartwright‐Hatton, 2004) *Social media addiction:* SMAS (Tutgun‐Ünal & Deniz, 2015)	Significant bivariate correlations between: ‐Positive beliefs about worry and SMAS relapse (*r* = 0.25***) and conflict sub‐dimensions (*r* = 0.24***) ‐Negative beliefs about thoughts concerning uncontrollability and danger and SMAS relapse (*r* = 0.16***) and conflict sub‐dimensions (*r* = 0.18***) ‐Cognitive confidence and SMAS mood regulation sub‐dimension ‐Compared to non‐addicts, SNS addicts obtained higher scores in: ‐Positive beliefs about worry; ‐Negative beliefs about thoughts concerning uncontrollability and danger; ‐Cognitive confidence; ‐Beliefs about the need to control thoughts	15
Casale et al. (2018)	Italy	PSNSU	Specific metacognitions to PSNSU	*n* = 579 undergraduates (*F* = 54.6%) *M*_age_ = 22.39 (*SD* = 2.82)	*Positive metacognitions about social media use:* Self‐report consisting of five items *Social Media Addiction:* BSMAS (Andreassen et al., 2016) *Fear of negative evaluation:* BFNE‐II (Carleton et al., 2007) *Fear of Missing Out*: FoMO scale (Przybylski et al., 2013) *Self‐presentational skills:* Social Skills Inventory (Riggio, 1986).	‐Significant bivariate correlations between PSNSU and positive metacognitions (*r* = 0.41***). ‐Positive metacognitions partially mediate the association between the fear of missing out and problematic SNSs use among both genders ‐Positive metacognitions fully mediate the association between fear of negative evaluation and self‐presentational skills, on the one hand, and PSNSU, on the other.	15
Ünal‐Aydin et al. (2021)	Turkey	PSNSU	Generic metacognitions	*n* = 861 high‐school students (*F* = 49.1%) *M*_age_ = 15.84 (*SD* = 0.96)	*Metacognitions:* MCQ‐C (Bacow et al., 2009) *PSNSU:* BSMAS (Andreassen et al., 2016)	‐Significant bivariate correlations between PSNSU and positive meta‐ worry (*r* = 0.22**), negative meta‐worry (*r* = 0.21**), beliefs about the need to control thoughts (*r* = 0.27**) and cognitive monitoring (*r* = 0.08*). Positive meta‐worry, negative meta‐ worry, beliefs about the need to control thoughts, and cognitive monitoring independently predicted the BSMAS total score controlling for daily SNS use.	17
Marino et al. (2019)	Italy	PFU	Generic metacognitions	Study 1 *n* = 271 high school students (*F* = 67.9%) *M*_age_ = 17.02 (*SD* = 1.56; age range = 14–20) Study 2 *n* = 336 high school students (*F* = 54.76%) *M*_age_ = 16.22 (*SD* = 1.41; age range = 14–20)	*Metacognitions*: MCQ‐30 (Wells & Cartwright‐Hatton, 2004) Problematic Facebook use: PFUS (Marino et al., 2017) *Attachment:* IPPA (Guarnieri et al., 2010) ECR‐RC (Brenning et al., 2014)	Study 1 Bivariate correlation between PFU and metacognitions total score *r* = 0.34*** Metacognitions (total score) had a mediating role between alienation towards parents and communication with father, and PFU. Study 2 Bivariate correlation between PFU and metacognitions total score *r* = 0.31*** Metacognitions (total score) mediated the links between avoidance towards both mother and father and PFU	15
Akbari et al. (2020)	Iran	PSU	Specific metacognitions to PSU	*n* = 618 community adults (*F* = 63.6%) *M*_age_ = 27.31 (*SD* = 8.95; age range: 15–67)	*Metacognitions:* MSUQ (Casale et al., 2020) *PSU:* SAS‐SV (Kwon et al., 2013) *Anxiety and Depression*: HADS (Zigmond & Snaith, 1983)	Bivariate correlations: n.r. Positive metacognitions about emotional and cognitive regulation, negative metacognitions about uncontrollability and cognitive harm, and positive metacognitions about social advantages, predicted smartphone addiction levels, independently of anxiety and depression.	14
Casale et al. (2020)	Italy	PSU	Specific metacognitions to PSU	*n* = 701 community participants (*F* = 66%) *M*_age_ = 28.08 (*SD* = 9.81; age range: 15–70)	*Metacognitions:* MSUQ (Casale et al., 2020) *Problematic Smartphone use:* PSUS (Kwon et al., 2013) *Anxiety and Depression*: HADS (Zigmond & Snaith, 1983)	‐Significant bivariate correlations between PSU and positive metacognitions about emotional and cognitive regulation (*r* = 0.23***), positive metacognitions about social benefits (*r* = 0.33***), negative metacognitions about uncontrollability and cognitive harm (*r* = 0.51***). ‐Positive metacognitions about emotional and cognitive regulation, positive metacognitions about social benefits, negative metacognitions about uncontrollability and cognitive harm were significant predictors of PSU, independently of anxiety and depression	16

Abbreviations. BFNE‐II = Brief Fear of Negative Evaluation‐II; BSMAS = Bergen Social Media Addiction Scale; DASS‐21 = Depression Anxiety Stress Scales; GPIUS‐2 = Generalized Problematic Internet Use Scale 2; HADS = Hospital Anxiety and Depression Scale; IGD = Internet Gaming Disorder; IAT = Internet Addiction Scale; IGDT = The Internet Gaming Disorder Test; MCQ‐30 = Metacognition Questionnaire‐30; MCQ‐C = Metacognitions Questionnaire for Children; MOGS = Metacognitions about Online Gaming Scale; MSUQ = Metacognitions about Smartphone use Questionnaire; nr = not reported; PFUS = Problematic Facebook Use Scale; PIU = Problematic Internet Use; PSNSU = Problematic Social Networking Sites Use; PSU = Problematic Smartphone Use; RMET = Children's Version of Reading the Mind in the Eye Test; SAS‐SV = Smartphone Addiction Scale‐Short Version; SMAS = Social Media Addiction Scale; SSI = Social Skills Inventory; SPIUT = Short Problematic Internet Use Test.

* *p* < 0.05.

** *p* < 0.01.

*** *p* < 0.001.

## 3 RESULTS

### 3.1 Demographics of the included studies

Thirteen studies focusing on metacognitions in technological addictions were published between 2018 and 2021 (Table 1). Four studies focused on PIU, three studies each focused on IGD and general PSNSU, one study addressed problematic Facebook use (PFU) and two studies investigated metacognitions in PSU. Sample sizes ranged between 180 (Hamidi & Ghasedi, 2020) and 861 participants (Ünal‐Aydin et al., 2021) and a mean of 504 participants. Age of participants ranged between 10 and 73 years old. Six samples were selected in Italy, three in Iran, two in Turkey and one each in Bosnia and Herzegovina and China. All the studies adopted a cross‐sectional design. Samples were predominantly mixed, consisting of both men and women, with the exception of two studies, which mainly involved men (Caselli et al., 2020; Marino et al., 2020). Only one study (Hamidi & Ghasedi, 2020) recruited a clinical sample. The majority of the included studies consisted of community adult samples (e.g., Akbari et al., 2020; Casale et al., 2018), whilst two studies focused on high school students (Aydın, Güçlü, et al., 2020; Marino et al., 2019), and one study involved early adolescents from middle schools (Marci et al., 2021). In the review, samples of adults, adolescents and children are addressed together because the types of problems associated with technological addictions do not differ between age groups (Kuss et al., 2021).

### 3.2 Measures of metacognitions

Twelve studies out of 13 used self‐report measures explicitly based on the S‐REF model to assess metacognitions, beside their focus on generic (*n* = 8) or rather specific metacognitions about technology (*n* = 5).

Generic metacognitions were assessed through the Metacognitions Questionnaire‐30 (MCQ‐30; Wells & Cartwright‐Hatton, 2004) or the Metacognitions Questionnaire—child version (MCQ‐C; Bacow et al., 2009). Two studies were conducted on IGD, and three studies each on PIU and PSNSU (one was specifically focused on PFU). The MCQ‐30 assesses the five dimensions of generic metacognitions, which have been found to be involved in the preservation of maladaptive coping ([1] positive beliefs about worry; [2] negative beliefs about thoughts concerning uncontrollability and danger; [3] beliefs about the need to control thoughts (also named beliefs about superstition, punishment and responsibility); [4] cognitive confidence [i.e., lack of confidence in memory and attention]; and [5] cognitive monitoring, also named cognitive self‐consciousness [i.e., the tendency to focus attention on thought processes]). The MCQ‐30 has a stable factor structure, as well as good reliability and validity across samples (e.g., Fergus & Bardeen, 2017). The MCQ‐C includes the parent version scales with the exception of the cognitive confidence domain owing to theoretical reasons. In the MCQ‐C, positive beliefs about worry are named positive meta‐worry, and negative beliefs about thoughts concerning uncontrollability and danger are named negative meta‐worry.

The measure used to assess specific metacognitions was based on the type of technological addiction. One study used the Metacognitions about Online Gaming Scale (MOGS; Spada & Caselli, 2017), and two studies used the Metacognitions about Problematic Smartphone Use Questionnaire (MSUQ; Casale et al., 2020). The MOGS and the MSUQ have been developed to assess specific positive and negative metacognitions about online gaming and smartphone use, respectively. The MOGS assesses positive metacognitions about online gaming (i.e., positive beliefs about the usefulness of engaging in online gaming as a cognitive and affective self‐regulation strategy) and negative metacognitions about the uncontrollability and dangers of online gaming. This self‐report measure showed good psychometric properties, including predictive and divergent validity (Spada & Caselli, 2017). Finally, the MSUQ has a three‐factor structure consisting of positive metacognitions concerning emotional and cognitive regulation (i.e., positive beliefs about the usefulness of smartphone use for emotional and cognitive regulation), positive metacognitions concerning social advantages (i.e., positive beliefs about the usefulness of smartphone use for regulating the fear of missing out and staying in touch) and negative metacognitions about uncontrollability and cognitive harm of smartphone use. Higher subtest scores are indicative of dysfunctional metacognitions in the given subdimension. The three‐factor structure of the MSUQ was confirmed through a confirmatory factor analysis and evidence of convergent and predictive validity was provided. Finally, one study (Casale et al., 2018) used a five‐item measure predisposed to assess specific metacognitions about PSNSU. Beyond the internal consistency, no information on the psychometric characteristics of this brief self‐report measure was given.

### 3.3 Generic metacognitions and technological addictions

Overall, bivariate correlations across studies showed a consistent pattern of results across PIU, IGD and PSNSU. Positive beliefs about or worry (also named positive meta‐worry) and negative beliefs about thoughts concerning uncontrollability and danger (also named negative meta‐worry) were consistently found to be positively associated with IGD (Aydın, Güçlü, et al., 2020), PSNSU (Balıkçı et al., 2020; Ünal‐Aydin et al., 2021) and PIU (Hashemi et al., 2020; Marci et al., 2021). The correlations were small across the studies, with the exception for a higher (i.e., moderate) associations found with PIU (Hashemi et al., 2020). Noteworthy, the sample recruited in this study had a mean age higher than the other samples.

Lack of confidence in memory and attention, and beliefs about the need to control thoughts, were found to be associated with higher scores on IGD measures among both adolescents (Aydın, Güçlü, et al., 2020) and university students (Zhang et al., 2020). Similarly, social networking site (SNS) problematic users showed significantly lower cognitive confidence and higher beliefs about the need to control thoughts than non‐SNS problematic users. Cognitive monitoring was the metacognitions with lower—albeit significant—associations with PIU and IGD (0.08 ≤ *r* ≤ 0.16), whilst SNS problematic users did not significantly differ with SNS non‐problematic users in this dimension.

Although the cross‐sectional design used by the studies does not allow for causal inferences, findings from studies positing a mediating role for generic metacognitions were consistent. It has been highlighted that generic metacognitions had a mediating role in the association between well‐known risk factors (e.g., negative affect) and PSNSU (Casale et al., 2018) and that they predict IGD over and beyond these factors (e.g., Zhang et al., 2020). None of the studies investigated generic metacognitions in PSU.

### 3.4 Specific metacognitions about technological addictions

A consistent pattern of results also emerged regarding the link between specific metacognitions and PIU, IGD, PSNSU and PSU. The association was stronger with negative metacognitions relative to positive metacognitions. For instance, bivariate coefficients of.051 and 0.64 were found with PSNSU (Casale et al., 2020) and IGD (Marino et al., 2020), respectively. Taken as a whole, the results highlight that specific metacognitions (i) had a mediating role in the association between negative affect and PIU (e.g., Caselli et al., 2020) and IGD (Marino et al., 2020) and (ii) predict PSU beyond depressive and anxiety symptoms (Akbari et al., 2020; Casale et al., 2020) and time spent using online gaming (e.g., Zhang et al., 2020).

## 4 DISCUSSION

The aim of the present study was to systematically review the current state of knowledge regarding metacognitions in technological addictions and to interpret these findings in relation to the metacognitive model of addictive behaviours (Spada et al., 2013; Spada, Caselli, et al., 2015). We reviewed 13 cross‐sectional studies examining metacognitions from four different technological addictions. In general, there is a paucity of studies examining metacognitions across these behaviours among treatment‐seeking samples. Consequently, conclusions drawn from the findings of the current review are necessarily tentative.

As a whole, the empirical evidence shows that people who engage in unregulated use of new technologies hold dysfunctional metacognitions, thus confirming the initial results based on a few studies mainly conducted on PIU highlighted by Hamonniere and Varescon (2018). Correlations between problematic use of new technologies and all the metacognitions, be they generic or specific, were significant and low to moderate. These results seem to further support previous arguments that metacognitions have a trans‐diagnostic nature (Spada, Caselli, et al., 2015). However, as already suggested (Wells & Matthews, 1996) and empirically highlighted (Sun et al., 2017), it is plausible that the type of metacognitions differ in the extent to which they are prominent in specific disorders. Hamonniere and Varescon (2018) have shown that negative beliefs about thoughts concerning uncontrollability and danger (negative meta‐worry), beliefs about the need to control thoughts, and a lack of cognitive confidence are the metacognitions most closely associated with addictive behaviours, whilst the tendency to focus attention on thought processes (i.e., cognitive monitoring) was less prominent. However, they also have highlighted that positive beliefs about worry are important with respect to technological addictions. In keeping with this perspective, and Hamonniere and Varescon's (2018) results, we found that negative meta‐worry, positive belies about worry (positive meta‐worry) and lack of cognitive confidence are positively associated with scores on technological addictions measures. Conversely, in accordance with this previous systematic review, we found that cognitive monitoring was the metacognition with the lowest—albeit significant—associations with PIU and IGD, and no significant differences were found on this dimension between SNSs problematic users and non‐problematic users. The belief that one's own thoughts need to be controlled, which is typical of OCD, and somewhat prevalent also in eating disorders and generalized anxiety disorders (Sun et al., 2017), might be less prominent in addictive behaviours, including technological addictions.

When it comes to metacognitions specific to addictive behaviours, we also found positive low to moderate associations between specific positive metacognitions and the four different technological addictions. The stronger the beliefs about the positive effects on emotions and cognitions of engaging in Internet, online games, social media and smartphone use, the higher the tendency to engage in these behaviours. In particular, a recent study (Caselli et al., 2020) found that positive metacognitions predict weekly online gaming hours, which, in turn, predict negative metacognitions. A strong indirect link was found between weekly online gaming hours and PIU via negative metacognitions about online gaming. Overall, these results provide further support to the notion that positive metacognitions play a central role in the pre‐engagement phase of an addictive behaviour because they motivate individuals to engage in addictive behaviour, whilst negative metacognitions increase the risk of a full‐fledged addictive behaviour (Spada et al., 2013; Spada & Wells, 2009).

Intriguing results also come from studies that have controlled for negative affect. Prior research in this field informs us that depression and anxiety levels need to be taken into account in research profiling metacognitions (Spada & Caselli, 2017). In fact, previous findings have consistently shown that negative affect predicts PIU (Pettorruso et al., 2020), IGD (e.g., Lin et al., 2020), PSNSU (Hou et al., 2019) and PSU (e.g., Vahedi & Saiphoo, 2018). On the one hand, various included studies revealed a mediating role of metacognitions in the association between negative affect and technological addictions (Marino et al., 2020). On the other hand, the present review highlights that metacognitions predict IGD and PSU beyond the negative affect (Akbari et al., 2020; Casale et al., 2020; Zhang et al., 2020). Overall, these findings show that metacognitions affecting technological addictions cannot be entirely traced back to anxiety and depressive symptoms, which is consistent with evidence of the mediating role of metacognitions when other psychosocial vulnerabilities were considered (see, e.g., Casale et al., 2018).

Unlike what has been done with drinking behaviour and nicotine dependence (see, Nikčević et al., 2017; Spada, Nikčević, et al., 2007), no studies to date have controlled for positive and negative expectancies when investigating the predictive role of metacognitions in technological addiction. As the metacognitive model of psychopathology states that the key markers of psychopathology are beliefs pertaining to the metacognitive rather than the cognitive domain (Wells, 2009), studies on the additional contribution of positive metacognitions beyond positive expectancies are essential to support the notion that positive metacognitions may play a causal role in the pre‐engagement and engagement phases of technological addictions. Positive expectancies have been defined as the anticipated reinforcement related to engaging in a specific behaviour (Rash & Copeland, 2008), whilst positive metacognitions have been defined as beliefs about the benefits of engaging in addictive behaviour as a means of cognitive and affective regulation. Previous studies (Nikčević et al., 2017; Spada, Moneta, & Wells, 2007) have shown a certain overlap between metacognitions and expectancies when it comes to beliefs regarding the effects of the behaviour on emotional self‐regulation (i.e., positive metacognitions). Both positive expectancies and metacognitions capture what are essentially motivations for engaging in a particular behaviour. Consequently, future research needs to address this point in the technology addiction field in order to add to the argument that there is a value in differentiating between positive metacognitions about technology use and positive expectancies concerning technology use.

We also want to reiterate the encouragement stated repeatedly (Hamonniere & Varescon, 2018; Sun et al., 2017) to adopt longitudinal designs in this field, in order to verify whether metacognitions play a role in the initiation and maintenance of the addictive behaviours, as suggested by metacognitive model of addictive behaviours. We also need to consider that when it comes to problematic technology use a spiral effect has also been hypothesized (Slater, 2007). It is fundamental to consider what the person is actually doing on social media or through his/her smartphone, because metacognitions that had led to technology use in the first place might be reinforced by the use of a particular type of media content.

Furthermore, no research has examined metacognitions about craving in the present field, although levels of craving appear to increase following smartphone and social media abstinence (e.g., Stieger & Lewetz, 2018; Wilcockson et al., 2019) and adults with IGD report boredom and the need for stimulation as consequences of an 84‐h Internet gaming abstinence (King et al., 2016). As previous research on smoking cessation has shown that people who tend to have a negative appraisal of their craving‐related thoughts present a greater risk of relapse after cessation (Nosen & Woody, 2014), it might be useful for future research to focus its attention on metacognitions about craving for Internet, social media, online games and smartphone use. We were not able to explore differences and similarities in metacognitions across different technological addictions given the few studies conducted on each phenomenon. We encourage future research to make comparisons between the four phenomena considered in the current review, to determine whether some metacognitions that may be more typical of a specific technological addiction might be useful from a clinical perspective as well. Moreover, future studies might want to include addictions in which over‐use of technology might be present without being a necessary component—that is, in some cases, the use of technology might simply be a vehicle or a means to access the object of the addiction. In fact, even if it seems reasonable to assume that online gambling, compulsive shopping and sex addiction would exist in the absence of technology and/or the Internet (Davis, 2001), the very distinction may not always be clear‐cut (Montag et al., 2021; Starcevic et al., 2020).

Despite the highlighted limitations, the current evidence gives initial support to the generalizability of the metacognitive model of addictive behaviours to technological addictions. Interventions that target metacognitions, like Metacognitive Therapy (Wells, 2013), could be beneficial for people showing problematic technology use, akin to what has been done for other addictive behaviours (Spada, Caselli, et al., 2015).

## CONFLICT OF INTEREST

None.

## Data Availability

Data sharing is not applicable to this article as no new data were created or analysed in this study.
